# Contribution of Pollinators to Seed Production as Revealed by Differential Pollinator Exclusion in *Clerodendrum trichotomum* (Lamiaceae)

**DOI:** 10.1371/journal.pone.0033803

**Published:** 2012-03-19

**Authors:** Ryota L. Sakamoto, Motomi Ito, Nobumitsu Kawakubo

**Affiliations:** 1 Graduate School of Arts and Sciences, The University of Tokyo, Tokyo, Japan; 2 Graduate School of Applied Biological Sciences, Gifu University, Gifu, Japan; University of Bristol, United Kingdom

## Abstract

A diverse assemblage of pollinators, such as bees, beetles, flies, and butterflies, will often visit a single plant species. However, evaluating the effect of several insects on fruit and seed production is difficult in plants visited by a variety of insects. Here, we analyzed the effect of three types of pollinators, *Papilio* spp., *Macroglossum pyrrhosticta*, and *Xylocopa appendiculata* on fruit and seed production in *Clerodendrum trichotomum* by using a flower visitor barrier experiment with nets of specific mesh sizes. As a result, fruit/flower and seed/ovule ratios were significantly lower under *Papilio* exclusion than under natural conditions. On the other hand, ratios were not significantly different between *Papilio* excluded and both *Papilio* and *M. pyrrhosticta* excluded treatments. Therefore, *Papilio* and *X. appendiculata* are effective pollinators, whereas *M. pyrrhosticta*, which was the most frequent visitor, of *C. trichotomum*, is not. From our observations of visiting behaviors, we believe that because *M. pyrrhosticta* probably promotes self- pollination, this species is a non-effective pollinator. This is the first study to separate and compare the contribution of various visitors to the reproductive success of a plant.

## Introduction

A diverse assemblage of pollinators will often visit a single plant species [1], [2]. Traditionally, pollination biologists have focused on the visitors of major flowers, i.e., those visitors that play large roles in the evolution of floral traits [3]–[Bibr pone.0033803-Bloch1] [5]. Thus, most research to date has focused on evaluating the relationship between the visits and effects of major visitors on seed production [6], [7].

Recent reports have suggested that both minor and major flower visitors can have an effect on reproductive success and the evolution of floral traits in plants that are visited by more than one insect species [8], [9]. Previous studies have estimated the efficiency of multiple pollinators in order to understand the contribution of different pollinators in generalist systems [10]–[Bibr pone.0033803-Young1] [Bibr pone.0033803-Conner1] [13]. For example, major and minor pollinators carry similar amounts of pollen on their bodies [14]. Further, infrequent flower visitors have been estimated to be efficient pollinators [15], [16]. Moreover, in *Gorteria* species, evolutionary shifts between major pollinators have not been the primary drive of floral variation [17]. In *Raphanus raphanistrum*, significantly different seed production resulted from non-significantly different flower visits [18]. These results suggest that we must quantitatively evaluate the effect of each pollinator on fruit and seed production in cases where flowers are visited by a diverse assemblage of pollinators.

Here, we conducted a flower visitor barrier experiment to estimate the effect of each pollinator on fruit and seed production. In many artificial bagging experiments, all visitors are removed to investigate self-pollination, self-compatibility, and the pollination efficiencies of individual visitors [19]–[Bibr pone.0033803-Miyake1] [Bibr pone.0033803-Morinaga1] [22]. In addition, to estimate the contributions of insects, wire mesh has been placed around *Aloe* species to exclude birds [23]–[Bibr pone.0033803-Wilson1] [25]. In contrast, we can quantitatively compare fruit and seed production with and without specific flower visitors because our new method excludes multiple visitors by covering plants with nets of specific mesh size.

In this study, the effect of a variety of pollinators on the reproductive success of *Clerodendrum trichotomum* is reported. *Clerodendrum trichotomum* is a suitable plant for this purpose, because it is visited by a variety of insect species and the different pollinators are estimated to have a different effect on reproductive success [20], [26]. We examined the flowering phenology of *C. trichotomum*, the frequency of insect visits, fruit and seed production following hand-pollination, the behaviors of insects, and fruit and seed production with and without visits from pollinators.

## Materials and Methods

### Plant species

*Clerodendrum trichotomum* Thunb is a widely distributed deciduous pioneer plant in Japan, China, Taiwan, and Korea. This plant occurs in roadside thickets, along forest margins, and in disturbed forests. Its flowers are protandrous, blooming at a height of 2–3 m from August to September, and have a pistil with four ovules [27], four stamens, and a white corolla with five lobes. The length of the petal is 11–13 mm and the corolla tube is 20–25 mm deep, from which the pistil and a stamen extend 25–30 mm.

Studies were conducted from 4 August to 10 November in 2008, from 6 August to 11 October in 2009, and from 2 August to 13 October in 2010 in Miyama forest, Gifu Prefecture, Japan (ca. 200 m alt; 35°57′1″N, 136°73′2″E). We randomly selected 64, 20, and 7 plants in 2008, 2009, and 2010, respectively.

### Observations of flowering phenology

To determine the period when a stigma can receive pollen grains, we observed flowering phenology from 14 August to 2 September in 2009. A digital camera (Pentax Optio W60; Hoya Corp., Tokyo, Japan) was used to automatically record images from 19 flowers on three plants at 10-min intervals. Flowers expected to open soon were selected and observed using the digital camera until the corolla fell off. We defined and recorded the following three stages: (1) the beginning of the staminate phase, when a bud started to open; (2) the beginning of the pistillate phase, when a pistil extended fully; and (3) flower closing, when the style faded or the corolla fell off.

### Observations of insect visits

We observed pollinator visits to *C. trichotomum* under natural conditions. In 2008, we counted the number of pollinator visits to six plants in a 4 m×4 m plot on 12, 19, and 26 August and on 2 and 9 September, to determine pollinator fauna. In 2009 and 2010, we focused on three insect groups (*Papilio helenus*, *P. dehaanii*, *P. protenor*, *P. macilentus*, and *P. maackii*; *Macroglossum pyrrhosticta*; and *Xylocopa appendiculata*) and counted the number of visits to two plants (47 inflorescences) on 11, 18, and 25 August and on 1 and 8 September and to three plants (43 inflorescences) on 9, 16, 23, and 30 August and 6 September in a 2 m×2 m plot, respectively. To observe their diurnal variations, we observed flower visitors over 11 recording cycles of 30 min separated by 1-h intervals from sunrise to sunset, for a total of 5.5 observation hours on all observation days. In 2009 and 2010, we tested whether the distribution of total pollinator visits during a day differed from a uniform distribution using a Kolmogorov–Smirnov test. All tests were conducted using R software (ver. 2.11.1).

### Hand-pollination experiments

We conducted hand-pollination experiments to examine the mating system of *C. trichotomum*. In 2009 and 2010, we randomly marked 104 and 151 flowers on 10 and 11 plants for the outcross pollination treatment and 67 and 142 flowers on 12 and 11 plants for the self-pollination treatment, respectively. We bagged emasculated flowers before anthesis to prevent pollination by insects. During the pistillate phase, we performed hand-pollination by covering the stigma surface completely with self- or outcross-pollen grains. After fruits had matured, we collected all fruits of each bagged flower and counted the number of seeds within each fruit. Given that a flower of *C. trichotomum* has four ovules [27], we were able to easily calculate the seed/ovule ratio. Next, we calculated the fruit/flower ratio and seed/ovule ratio for each flower. The differences in the fruit/flower ratio and seed/ovule ratio between the outcross pollination and self-pollination treatments were tested using the Mann–Whitney *U*-test.

### Flower visitor barrier experiments

We analyzed the effects of pollination by *Papilio* spp. (*Papilio helenus*, *P. dehaanii*, *P. protenor*, *P. macilentus*, and *P. maackii*), *M. pyrrhosticta*, and *X. appendiculata* on seed production in *C. trichotomum* by using net covering experiments. To examine the effects of *Papilio* spp., we conducted experiments that excluded visits from *Papilio* spp. These entailed covering three and two plants with a 100-mm net in 2009 and 2010, respectively. We concluded that there were no morphological or behavioral differences among the *Papilio* spp. as pollinators. Additionally, to examine the effects of *M. pyrrhosticta* and *X. appendiculata*, we conducted experiments that excluded visits from both *Papilio* spp. and *M. pyrrhosticta*. These entailed covering two plants with a 25-mm net in 2010. The nets were made using green polyethylene cords and were placed without contacting the surfaces of inflorescences. In our preliminary experiment, we tested nets of a variety of mesh sizes and found that the 100-mm and 25-mm mesh nets were the most effective for excluding *Papilio* spp. and *M. pyrrhosticta*.

We attempted to examine the behaviors of flower visitors inside the nets. To do so, we observed 23 inflorescences on five plants covered with a 100-mm net, 53 inflorescences on two plants covered with a 25-mm net, and 48 inflorescences on two uncovered plants, for a total of 14 h of video recordings for each experiment. Video recording was conducted hourly from 09:00 to 15:00 on 10–17 August in 2010. We counted the number of approaches to inflorescences and the occurrence of visits on one inflorescence. In addition, we measured the amount of time spent drinking nectar per visit, which is one of the indices of pollination efficiency [28]. Differences in the frequency of approach per hour, the frequency of visits per approach on one inflorescence per hour, and the time spent drinking nectar during one visit per hour among insect groups and treatments were tested by Tukey's multiple comparison test.

To examine the effects of the three pollinator groups on fruit and seed production, we conducted the flower visitor barrier experiment using 100-mm nets on three and two plants (with 20 and 5 inflorescences) in 2009 and 2010, respectively, and using 25-mm nets on two plants (with 5 inflorescences) in 2010. We also observed four plants with 16 and 7 inflorescences in 2009 and 2010, respectively, under natural condition as control experiments. Upon maturation, we collected all fruits from covered and uncovered plants and calculated the fruit/flower and seed/ovule ratios for each flower.

The fruit/flower and seed/ovule ratios were compared among the three treatments (outcross pollination, natural conditions, and *Papilio* spp. excluded) in 2009 and four treatments (outcross pollination, natural conditions, *Papilio* spp. excluded, and both *Papilio* spp. and *M. pyrrhosticta* excluded) in 2010. We tested the effects of treatment on the fruit/flower and seed/ovule ratios using generalized linear mixed models (GLMM) followed by binomial and Poisson distributions, respectively, where treatments were considered as fixed factors and individual plant as random factors. We constructed models from a combination of three treatments as fixed factors in 2009, which are (1) outcross pollination vs. natural conditions vs. *Papilio* spp. excluded; (2) outcross pollination vs. the sum of natural conditions and *Papilio* spp. excluded; (3) the sum of outcross pollination and *Papilio* spp. excluded vs. natural conditions; (4) the sum of outcross pollination and natural conditions vs. *Papilio* spp. excluded; and (5) the sum of all three treatments. Model fit was judged using the Akaike information criterion (AIC). If an AIC value in the model showed that the sum of all three treatments was the smallest, it was assumed that there were no differences in the fruit/flower and seed/ovule ratios among treatments. In 2010, we applied this method equally for four treatments and produced 15 models.

## Results

### Flowering phenology of *C. trichotomum*

**Figure 1:**
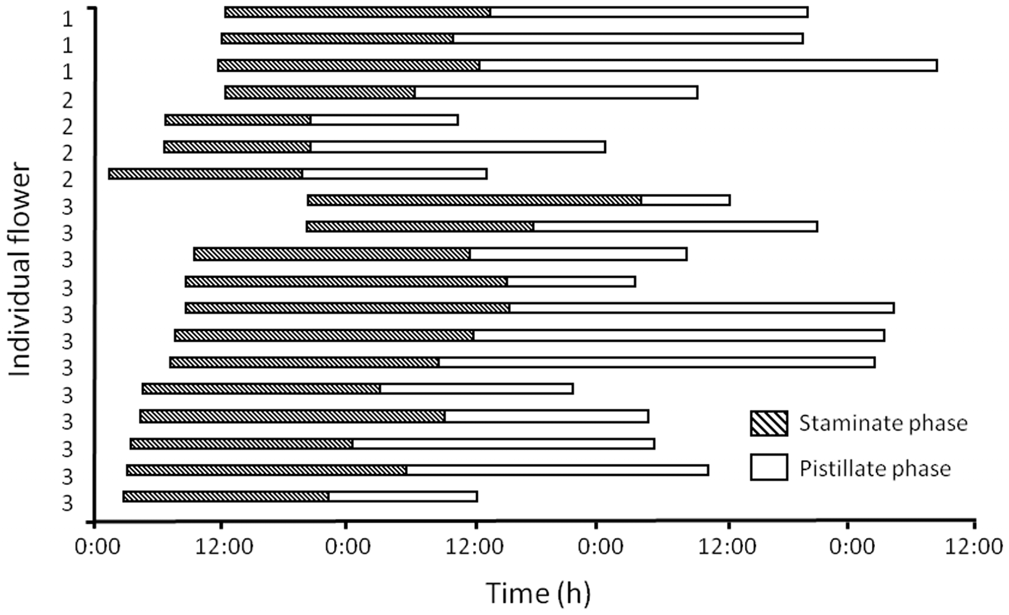
Flowering phenology of 19 flowers on three *C. trichotomum* plants. The hatched bar indicates the staminate phase and the white bar represents the pistillate phase. Numbers denote individual plants.

The flowers of *C. trichotomum* are protandrous and undergo two distinct phases. The staminate phase begins when the flowers open, and this is followed by the pistillate phase. Among the 19 flowers observed, the longevity of flowers varied from 27.8 to 68.5 h (Fig. 1; mean ± standard deviation (SD): 49.4±12.1). During anthesis, the staminate and pistillate phases lasted 13.8–31.7 h (23.7±5.4) and 8.3–43.5 h (25.7±10.3), respectively. The time at which the flowers opened varied and transition from the staminate to pistillate phase occurred approximately 1 d after the flowers opened. The longevity of the pistillate phase was not fixed.

### Fauna of flower visitors

In 2008, the flowers of *C. trichotomum* were visited by at least 12 species (comprising 8 genera) of insects, for a total of 663 recorded visits (Table 1). Of these, 41% were from *Papilio* spp. (*P. helenus*, *P. dehaanii*, *P. protenor*, *P. macilentus*, and *P. maackii*), 46% were from *M. pyrrhosticta*, and 8% were from *X. appendiculata*. These insects accounted for 95% of the total visits. In 2009 and 2010, we focused on these three insect groups and recorded 86 and 110 visits by *Papilio* spp. (26% and 17%), 85 and 429 visits by *M. pyrrhosticta* (26% and 66%), and 158 and 107 visits by *X. appendiculata* (48% and 17%), respectively.

**Table 1:** Frequencies of flower visitors in *C. trichotomum*.

Flower visitor species	2008 (16 m^2^)		2009 (4 m^2^)		2010 (4 m^2^)	
*Papilio*	266	[41%]	86	[26%]	110	[17%]
*P. helenus*	155		50		50	
*P. dehaani*	91		28		55	
*P. protenor*	17		6		5	
*P. macilentus*	2		0		0	
*P. maackii*	1		2		0	
*Macroglossum pyrrhosticta*	312	[46%]	85	[26%]	429	[66%]
*Theretra oldenlandiae*	12	[2%]	-		-	
*Pelopidas mathias*	3	[0.4%]	-		-	
*Pyralidae* sp.	2	[0.2%]	-		-	
*Xylocopa appendiculata*	53	[8%]	158	[48%]	107	[17%]
*Amegilla quadrifasciata*	11	[2%]	-		-	
*Vespa simillima*	4	[0.4%]	-		-	
Total	663	[100%]	329	[100%]	646	[100%]

Observations were conducted in a 4 m×4 m plot in 2008 and in 2 m×2 m plots in 2009 and 2010. Total time of observation per year was 27.5 h.

**Figure 2:**
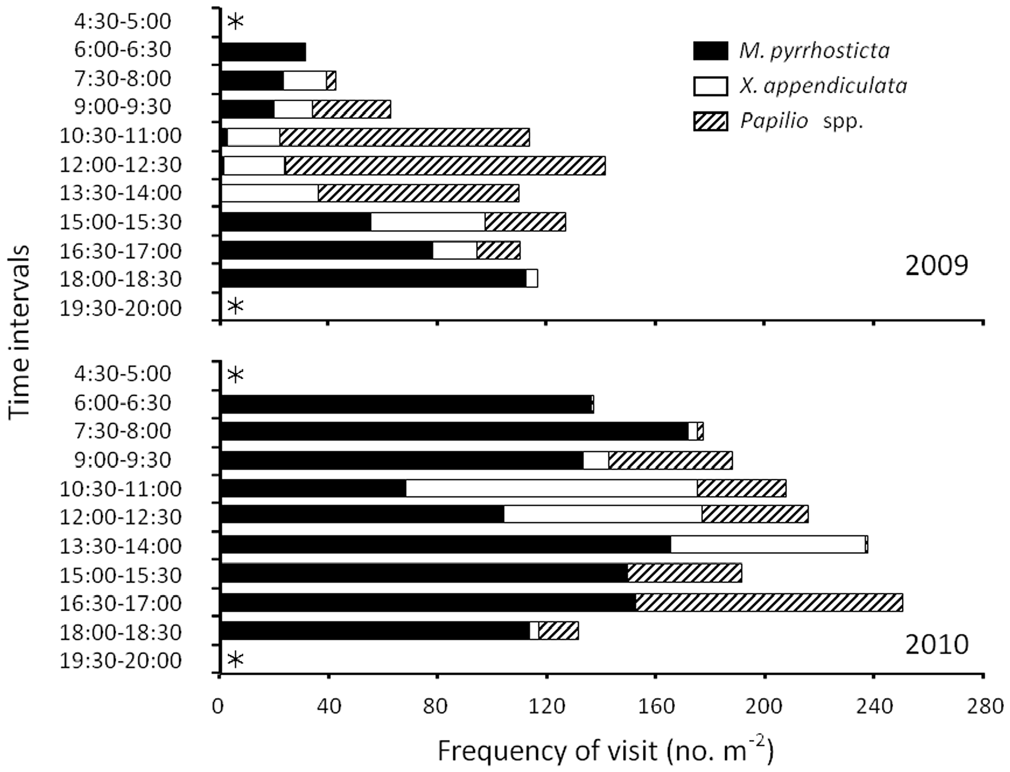
The frequency of visits per area for *M. pyrrhosticta*, *X. appendiculata*, and *Papilio* spp. in 2009 and 2010. Each period represents a total of five observations in one flowering season. Asterisks indicate that no flower visitors were observed.

Insect visits were observed from 06:00 to 18:30 in both years (Fig. 2). The frequencies of visits per area varied between years, although the same trends were observed in both years (Fig. 2). In 2009 and 2010, the frequencies of visits in a day deviated significantly from a uniform distribution for *Papilio* spp. (Kolmogorov–Smirnov test: *D* = 0.45, *P* = 0.03; *D* = 0.43, *P* = 0.03) and *M. pyrrhosticta* (*D* = 0.42, *P* = 0.05; *D* = 0.40, *P* = 0.05), respectively. For *X. appendiculata*, the frequency of visits did not deviate from a uniform distribution in 2009 (*D* = 0.27, *P* = 0.46), but did deviate in 2010 (*D* = 0.60, *P*<0.01). *M. pyrrhosticta* visited more often in the mornings and evenings compared to during the day. In contrast, *X. appendiculata* visited only in the daytime and *Papilio* spp. visited from early morning to late evening.

### Seed and fruit production in outcross pollination and self-pollination treatments

In the self-pollination treatment, few fruits and seeds were produced in both 2009 and 2010 (Table 2). In the outcross pollination treatment, not all flowers and ovules matured into fruits and seeds. The fruit/flower ratio was approximately 2- and 30-fold higher in the outcross pollination treatment than in the self-pollination treatment in 2009 and 2010, respectively. Additionally, the seed/ovule ratio was approximately 3- and 70-fold higher in the outcross pollination treatment than in the self-pollination treatment in 2009 and 2010, respectively. We observed significant differences in the fruit/flower ratio (Mann–Whitney *U*-test: outcross N = 104; self N = 67, *U* = 4478.5, *P*<0.01 and outcross N = 151; self N = 142, *U* = 13699, *P*<0.01) and the seed/ovule ratio (outcross N = 104; self N = 67, *U* = 4576, *P*<0.01 and outcross N = 151; self N = 142, *U* = 13714, *P*<0.01) between the hand-pollination treatments in 2009 and 2010, respectively (Table 2).

**Table 2:** Fruit and seed production in hand-pollination experiments in *C. trichotomum*.

	2009		2010	
	Cross (N = 104)	Self (N = 67)	Cross (N = 151)	Self (N = 142)
Fruit-flower ratio	0.45±0.05	0.22±0.05**	0.28±0.04	0.01±0.01**
Seed-ovule ratio	0.29±0.04	0.10±0.03**	0.15±0.02	0.002±0.002**

mean ± SE,

** : *P*<0.01.

Cross:Outcross pollination treatment, Self:Self-pollination treatment.

The fruit/flower and seed/ovule ratios were analyzed using the Mann-Whitney *U*-test.

### Effects of flower visitor barrier on pollinator behaviors and reproductive successes

Visits from *Papilio* spp. and both *Papilio* spp. and *M. pyrrhosticta* to the flowers were blocked completely by the flower visitor barriers using 100-mm and 25-mm nets, respectively (Table 3). For *M. pyrrhosticta*, there were no differences in the frequency of approach, the frequency of visit, and time spent drinking nectar between natural conditions and flower visitor barriers using a 100-mm net (Tukey's multiple comparison tests). Further, in *X. appendiculata*, there were no significant differences in the frequency of approach, the frequency of visit, and time spent drinking nectar among treatments. Under natural conditions, the frequency of approach and frequency of visit of *Papilio* spp. were significantly lower than those of *M. pyrrhosticta*. Furthermore, under natural conditions, the time spent drinking nectar for *X. appendiculata* was approximately 10-fold longer than that of *Papilio* spp. and *M. pyrrhosticta*.

**Table 3:** Frequencies and duration of insect group behaviors under natural conditions and under the flower visitor barriers per hour in 2010.

	*Papilio* spp.			*M. pyrrhosticta*			*X. appendiculata*		
	Control	100-mm net	25-mm net	Control	100-mm net	25-mm net	Control	100-mm net	25-mm net
Frequency of approach	0.38±0.22^b^	0^c^	0^c^	1.42±0.38^a^	1.46±0.39^a^	0^c^	0.84±0.25^ab^	0.90±0.31^ab^	1.04±0.27^ab^
Frequency of visit	1.29±0.73^b^	0^c^	0^c^	5.28±1.41^a^	3.49±0.93^a^	0^c^	2.45±0.75^ab^	2.39±0.83^ab^	3.56±1.03^a^
Nectar drinking time (sec.)^a^	0.27±0.7^b^	0^c^	0^c^	0.27±0.07^b^	0.25±0.07^b^	0^c^	2.24±0.60^a^	1.91±0.51^a^	2.32±0.62^a^

mean ± SE.

Control: natural conditions; 100-mm net: flower visitor barrier with a 100-mm mesh; 25-mm net: flower visitor barrier with a 25-mm mesh.

Different letters indicate significant differences in behaviors among the three insect groups using Tukey's multiple comparison test (*P*<0.05).

Observations were conducted over 14 h. Differences in behaviors among the three treatments were analyzed using Tukey's multiple comparison test.

**Figure 3:**
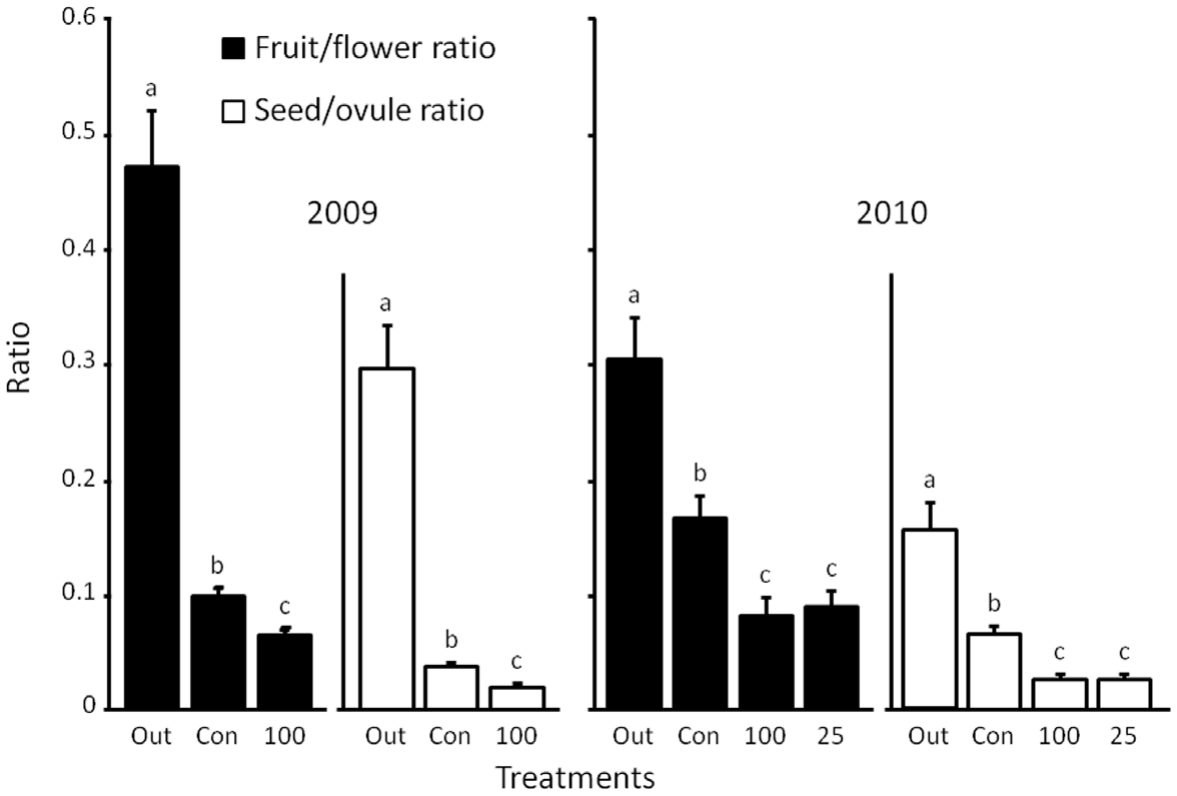
Comparison of the fruit/flower and seed/ovule ratios among the three treatments in 2009 and the four treatments in 2010. Letters indicate differences in the fruit/flower and seed/ovule ratios as determined by AICs depending on GLMMs. “Out” indicates the outcross-pollination treatment, “Con” denotes the control experiment, “100” denotes the flower visitor barrier experiment with a 100-mm mesh, and “25” refers to the flower visitor barrier experiment with a 25-mm mesh.

We compared the fruit/flower and seed/ovule ratios among treatments (Fig. 3). GLMM analysis revealed that the fruit/flower and seed/ovule ratios were different among the three treatments on the AIC in 2009 (Table S1; minimum AICs were 2303 and 722.9 when values of the three treatments were different in the fruit/flower and seed/ovule ratios; maximum AICs were 2317 and 886.9 when the values of the three treatments were the same, respectively). In 2010, GLMM analysis also revealed that the fruit/flower and seed/ovule ratios were different among outcross pollination, natural condition, and flower visitor barriers, but not different between the size of meshes on the AIC in 2010 (minimum AICs were 935 and 268.6 when outcross pollinations vs. natural condition vs. the sum of *Papilio* spp. excluded and both *Papilio* spp. and *M. pyrrhosticta* excluded; maximum AICs were 947.1 and 466.1 when the values of the four treatments were the same, respectively).

## Discussion

Flower visitor barrier experiments successfully blocked *Papilio* spp. and *M. pyrrhosticta* from visiting flowers, but did not significantly exclude *X. appendiculata* (Table 3). There were significant differences in the fruit/flower and seed/ovule ratios between outcross pollination and natural conditions (Fig. 3). This indicates that the reproductive success of *C. trichotomum* is limited, because an insufficient amount of pollen is deposited. Therefore, if flower visitor barriers exclude pollinators, the number of deposited pollen grains decreases further. As a result, we were able to estimate decreases in fruit/flower and seed/ovule ratios as contributions from pollinators. Although some studies have used barriers to exclude pollinators and determine the contribution of the pollinator [23]–[Bibr pone.0033803-Wilson1] [25], some of our barriers excluded multiple pollinators. To the best of our knowledge, this is the first study to successfully use different mesh sizes to differentially exclude pollinators and hence determine the relative contribution of these insects.

### Mating system and flower visitors in *C. trichotomum*

The blooming times of *C. trichotomum* flowers varied, even on the same individual (Fig. 1). The pistillate phase, during which flowers are able to receive pollen grains, continued throughout both the daytime and nighttime (Fig. 1). Therefore, *C. trichotomum* probably receives pollen grains throughout a 24-h period. In addition, because the fruit/flower and seed/ovule ratios in the self-pollinating treatment were significantly lower than in the outcross pollination treatment (Table 2), *C. trichotomum* requires pollinators for reproductive success. Furthermore, not all ovules matured to seeds, even if flowers received pollen grains in the outcross pollination treatment (Table 2). We accordingly that *C. trichotomum* may experience resource limitation.

Our observations of flower visitors indicated that *C. trichotomum* is visited by a variety of insect species (Table 1), the visiting frequencies of which vary throughout the day (Fig. 2). However, fruit and seed production under natural conditions were significantly lower than those in the artificial outcross pollination treatment (Fig. 3). Thus, *C. trichotomum* appears to be under pollinator limitation because pollen grains that are attached to pollinators were insufficient for seed production despite the fact that several pollinators visited the flowers.

Reproductive success is dependent on the timing of when flowers can receive pollen grains [29], [30] and when insects visit the flowers [20], [22]. For *C. trichotomum*, we determined when flowers were able to receive pollen grains (Fig. 1) and when the specific insect species visited the flowers (Fig. 2). The findings demonstrated that all flower visitors were potential pollinators and that variations in flowering time could be an adaptation to variations in visiting peak time among pollinator insects.

### Differences in pollination contribution among flower visitors

On the basis of the results of the flower visitor barrier experiments, *Papilio* spp. and *X. appendiculata* contribute to fruit and seed production in *C. trichotomum* (Fig. 3). In addition, because the combined visits of *Papilio* spp., *M. pyrrhosticta*, and *X. appendiculata* accounted for 95% of the total visits (Table 1), we believe that fruits and seeds that were produced under both the *Papilio* spp. and *M. pyrrhosticta* exclusion treatments might demonstrate pollination by *X. appendiculata*. Furthermore, because the flowers of *C. trichotomum* are entomophilous, we believe that the effect of wind pollination is small. We will clarify in future studies whether the pollen grains are carried by wind.

The fruit/flower and seed/ovule ratios under the flower visitor barrier treatment using a 100-mm mesh were significantly lower than under natural conditions (Fig. 3). These differences indicate the contribution of *Papilio* spp. to seed production. Previous studies have reported both high [12] and low [31]–[Bibr pone.0033803-Weiss1] [33] pollination efficiencies in butterflies. In addition, some studies have suggested that most butterflies are nectar robbers [34], [35]. However, these studies did not evaluate the effect of butterflies on the reproductive success of plants.

There were no differences in the fruit/flower and seed/ovule ratios between flower visitor barrier treatments which excluded *Papilio* spp. (100-mm mesh) and those which excluded *Papilio* spp. and *M. pyrrhosticta* (25-mm mesh) (Fig. 3). These results show that *M. pyrrhosticta* did not contribute to fruit and seed production, even though this species had the highest visitation frequency (Table 1; Table 3). An understanding of the behaviors of flower visitors is essential to evaluate the reproductive success of plants [36], [37]. Given that more visits on one inflorescence enables geitonogamous pollination [38], [39], the high frequency of visits of *M. pyrrhosticta* probably promotes self-pollination. Therefore, *M. pyrrhosticta* may not be an efficient pollinator.

On the other hand, *X. appendiculata* contributed to fruit and seed production (Fig. 3), although these insects primarily appeared to be nectar robbers (Sakamoto, personal observation; i.e., they drilled holes in corolla tubes [40]). Many studies have reported that nectar robbers are not effective pollinators and that they reduce the reproductive success of plants [41]–[Bibr pone.0033803-Gill1] [43], but see [44]. If *X. appendiculata* contacted the sexual organs of flowers before they drilled holes in the corolla tubes, they may have acted as efficient pollinators even if they were nectar robbers. In future studies, we will clarify the timing of the deposition and removal of pollen grains. Additionally, the time spent drinking nectar for *X. appendiculata* was significantly longer than that for *Papilio* spp. and *M. pyrrhosticta* (Table 3). These differences in the time spent drinking nectar may affect the receipt and deposit of pollen grains [45], [46].

Our field experiments successfully excluded multiple flower visitors without affecting the behaviors of non-excluded flower visitors. Nonetheless, we could not clarify the contributions and behaviors of pollinators that infrequently visited flowers, such as the nocturnal hawk moth *Theretra oldenlandiae*. We will evaluate the contributions of all pollinators in a future study by conducting additional barrier experiments. In addition, subsequent visits by pollinators can have different effects on pollen transfer depending on whether they are conducted by the same or different individuals. In a future study, we will also identify individual insects and compare the contributions between visits by the same or different insects by studying visitation behaviors (e.g., flight range and the frequency of geitonogamy). Moreover, in the present study, we limited our focus to seed production, which is an indication of female reproductive success, and did not discuss the transfer of pollen grains, which is an indicator of male reproductive success. In future studies, we should assess the contributions of these three pollinators on both female and male reproductive success.

## Supporting Information

Table S1 AIC values from GLMM analyses that included different combinations of treatments as a fixed factor. We created 5 and 15 models from 3 and 4 treatments in 2009 and 2010, respectively. Models were considered substantially different if the difference in their AICs values was greater than 2 [47].
(DOC)
