# Supplementary material for: Contribution of Pollinators to Seed Production as Revealed by Differential Pollinator Exclusion in Clerodendrum trichotomum (Lamiaceae)
Source: PLoS One. 2012 Mar 19;7(3):e33803. doi: 10.1371/journal.pone.0033803 (PMC3307763; doi:10.1371/journal.pone.0033803)
Supplement: Table S1 — AIC values from GLMM analyses that included different combinations of treatments as a fixed factor. We created 5 and 15 models from 3 and 4 treatments in 2009 and 2010, respectively. Models were considered substantially different if the difference in their AICs values was greater than 2 [47]. (DOC) [file pone.0033803.s001.doc]

| **Table S1** AIC values from GLMM analyses that included different combinations of treatments as a fixed factor. We created 5 and 15 models from 3 and 4 treatments in 2009 and 2010, respectively. Models were considered substantially different if the difference in their AICs values was greater than 2 [47]. | | |
| --- | --- | --- |
| 2009 | | |
| Combinations of treatments as fixed factor | Fruit/flower ratio | Seed/ovule ratio |
| Cross vs. Control vs. 100-mm net | 2303^+^ | 722.9^+^ |
| Cross vs. Control and 100-mm net | 2305 | 889.2^++^ |
| Control vs. Cross and 100-mm net | 2319^++^ | 742.1 |
| Cross and Control vs. 100-mm net | 2318 | 741.3 |
| Cross, Control, and 100-mm net | 2317 | 886.9 |
|  |  |  |
| 2010 | | |
| Combinations of treatments as fixed factor | Fruit/flower ratio | Seed/ovule ratio |
| Cross vs. Control vs. 100-mm net vs. 25-mm net | 937.2 | 270.8 |
| Cross vs. Control, 100-mm net, and 25-mm net | 938.5 | 468.7^++^ |
| Cross, 100-mm net, and 25-mm net vs. Control | 949.1 | 283.6 |
| Cross, Control, and 25-mm net vs. 100-mm net | 943.6 | 281 |
| Cross, Control, and 100-mm net vs. 25-mm net | 949.1 | 281.8 |
| Cross and Control vs. 100-mm net and 25-mm net | 939.1 | 273.9 |
| Cross and 100-mm net vs. Control and 25-mm net | 949.1 | 282 |
| Cross and 25-mm net vs. Control and 100-mm net | 944.8 | 281.9 |
| Cross and Control vs. 100-mm net vs. 25-mm net | 940.3 | 275.9 |
| Cross and 100-mm net vs. Control vs. 25-mm net | 951.1^++^ | 283 |
| Cross and 25-mm net vs. Control vs. 100-mm net | 944.8 | 281.9 |
| Cross vs. Control and 100-mm net vs. 25-mm net | 940.5 | 272.9 |
| Cross vs. Control and 25-mm net vs. 100-mm net | 937.5 | 272.7 |
| Cross vs. Control vs. 100-mm net and 25-mm net | 935^+^ | 268.6^+^ |
| Cross, Control, 100-mm net and 25-mm net | 947.1 | 466.1 |
| Cross: outcross pollination; Control: natural conditions; 100-mm net: flower visitor barrier with a 100-mm mesh; 25-mm net: flower visitor barrier with a 25-mm mesh. | | |
| The value of AICs revealed by GLMM analyses, ^+^: min AICs, ^++^: max AICs | | |
